# *N*,*N*-Di­phenyl-4-(1*H*-pyrrolo[1,2-*f*][1,10]phenanthro­lin-2-yl)­aniline ethanol monosolvate

**DOI:** 10.1107/S1600536813008477

**Published:** 2013-04-10

**Authors:** Jun-Shan Luo, Yan-Yan Zhang, Zhao-Di Liu, Yu-Peng Tian

**Affiliations:** a Deparment of Chemistry, Anhui University, Hefei 230601, People’s Republic of China; b Key Laboratory of Functional Inorganic Materials, Chemistry, Hefei 230601, People’s Republic of China

## Abstract

The title compound, C_32_H_21_N_4_·C_2_H_5_OH, crystallized as an ethanol monosolvate. In the mol­ecule of this phenanthroline derivative, the pyridine rings are almost coplanar, making a dihedral angle of 1.54 (13)°. The tri­phenyl­amine group, introduced as an electron donor, shows a propeller-type structure, and the dihedral angles between the benzene rings are 68.71 11), 63.92 (16) and 70.81 (15)°. In the crystal, the phenanthroline mol­ecules are linked *via* the solvent mol­ecule by N—H⋯O, O—H⋯N and C—H⋯O hydrogen bonds, leading to the formation of zigzag chains propagating along [010]. These chains are linked *via* C—H⋯N hydrogen bonds, forming undulating two-dimensional networks extending in the *a*- and *b*-axis directions.

## Related literature

For background to imidazo[4,5-*f*]-1,10-phenanthroline compounds, see: Li *et al.* (2012 ▶). For metal complexes and binding studies, see: Ma *et al.* (2009 ▶); Xu *et al.* (2012 ▶); Zheng *et al.* (2013 ▶). For the crystal structures of related compounds, see: Sun *et al.* (2009 ▶); Eseola *et al.* (2012 ▶); Bhat *et al.* (2011 ▶).
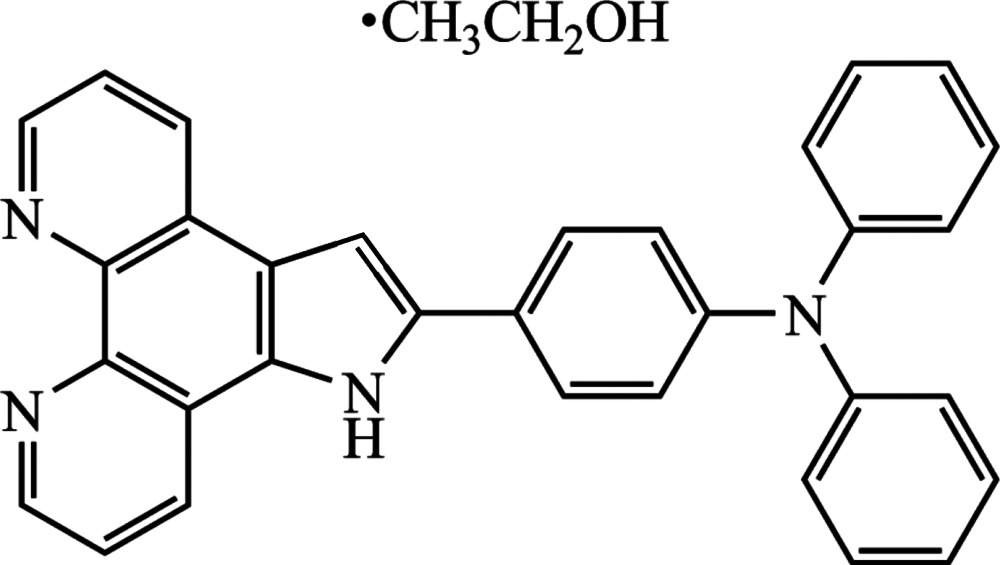


## Experimental

### 

#### Crystal data

C_32_H_21_N_4_·C_2_H_6_O
*M* *_r_* = 507.60
Monoclinic, 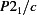

*a* = 9.716 (4) Å
*b* = 10.690 (4) Å
*c* = 27.017 (10) Å
β = 92.317 (4)°
*V* = 2803.8 (18) Å^3^
*Z* = 4
Mo *K*α radiation
μ = 0.07 mm^−1^
*T* = 296 K
0.20 × 0.20 × 0.10 mm

#### Data collection

Bruker APEXII CCD diffractometer
Absorption correction: multi-scan (*SADABS*; Bruker, 2007 ▶) *T*_min_ = 0.985, *T*_max_ = 0.993
20210 measured reflections
5216 independent reflections
3870 reflections with *I* > 2σ(*I*)
*R*_int_ = 0.024

#### Refinement

*R*[*F* ^2^ > 2σ(*F* ^2^)] = 0.065
*wR*(*F* ^2^) = 0.210
*S* = 1.02
5216 reflections
340 parameters
6 restraints
H atoms treated by a mixture of independent and constrained refinement
Δρ_max_ = 0.51 e Å^−3^
Δρ_min_ = −0.50 e Å^−3^

### 

Data collection: *APEX2* (Bruker, 2007 ▶); cell refinement: *SAINT* (Bruker, 2007 ▶); data reduction: *SAINT*; program(s) used to solve structure: *SHELXS97* (Sheldrick, 2008 ▶); program(s) used to refine structure: *SHELXL97* (Sheldrick, 2008 ▶); molecular graphics: *SHELXTL* (Sheldrick, 2008 ▶) and *PLATON* (Spek, 2009 ▶); software used to prepare material for publication: *SHELXTL*.

## Supplementary Material

Crystal structure: contains datablock(s) I, global. DOI: 10.1107/S1600536813008477/su2573sup1.cif

Structure factors: contains datablock(s) I. DOI: 10.1107/S1600536813008477/su2573Isup2.hkl

Supplementary material file. DOI: 10.1107/S1600536813008477/su2573Isup3.cml

Additional supplementary materials:  crystallographic information; 3D view; checkCIF report

## Figures and Tables

**Table 1:** Hydrogen-bond geometry (Å, °)

*D*—H⋯*A*	*D*—H	H⋯*A*	*D*⋯*A*	*D*—H⋯*A*
N3—H3*N*⋯O1	0.94 (3)	1.84 (3)	2.777 (3)	175 (2)
O1—H1*O*1⋯N1^i^	0.82	2.15	2.819 (3)	139
O1—H1*O*1⋯N2^i^	0.82	2.38	3.087 (3)	145
C16—H16⋯O1	0.93	2.54	3.419 (4)	157
C3—H3⋯N2^i^	0.93	2.59	3.310 (4)	135

Symmetry code: (i) 
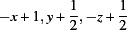
.

**Fig. 1.**
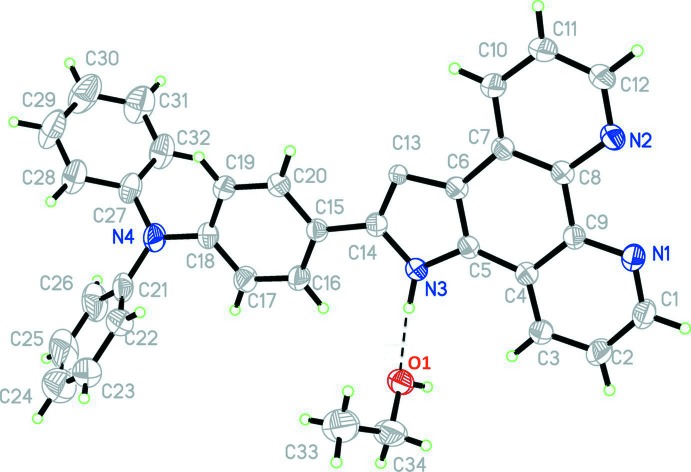
The molecular structure of the title molecule, with atoms labelling. The displacement ellipsoids are drawn at the 30% probability level. The N-H···O hydrogen bond is shown as a dashed line (see Table 1 for details).

**Fig. 2.**
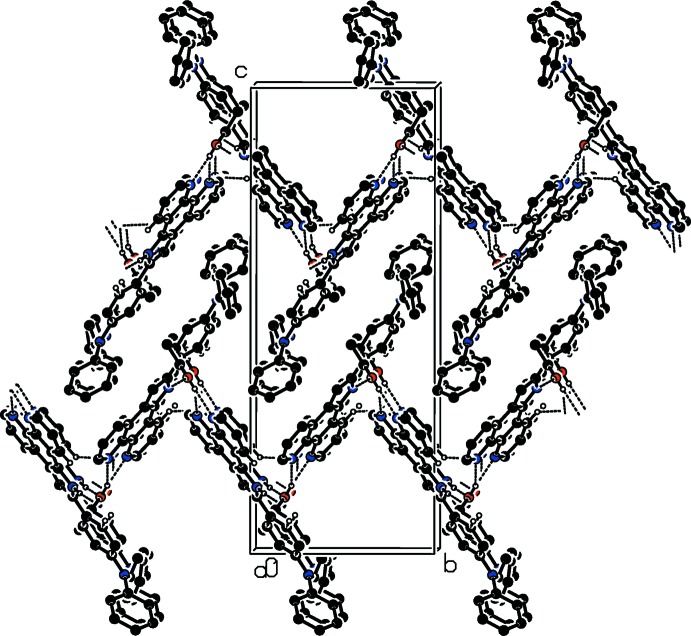
A view along the a axis of the crystal packing of the title compound, showing the various hydrogen bonds as dashed lines [see Table 1 for details; H-atoms not involved in these interactions have been omitted for clarity].

## supplementary crystallographic information

### Comment

1*H*-imidazo [4.5 - *f*] [1,10] phenanthroline derivatives have good biocompatibility and coordination ability (Li *et al.*, 2012). Such compounds are often used to coordinate with various metal ions, and their binding with DNA to achieve anti-cancer anti-tumor activity has been studied (Ma *et al.*, 2009; Xu *et al.*, 2012 ; Zheng *et al.*, 2013). The crystal structures of similar compounds have been reported (Sun *et al.*, 2009; Eseola *et al.*, 2012; Bhat *et al.*, 2011). As a part of ongoing study of this type compound, here we report on the crystal structure of the title compound.

The molecular structure of the title compound is illustrated in Fig. 1. It is comprised of a phenanthroline derivative and an ethanol solvent molecule (Fig. 1 and Table 1). The triphenylamine group, introduced as an electron donor, shows the propeller structure, with the dihedral angles between the benzene rings, (C15-C20), (C21-C26) and (C27-C32), being 68.71 (11), 63.92 (16) and 70.81 (15) °, respectively.

The two pyridine rings of the phenanthroline group are almost coplanar, with a dihedral angle of 1.54 (13)°. The mean plane of imidazo-phenanthroline group (N1-N3/C1-C14; r.m.s. 0.002) is inclined to the benzene ring to which it is attached by 7.63 (9)°.

In the crystal, the phenanthroline molecules are linked via the solvent molecule by N-H···O, O-H···N and C-H···O hydrogen bonds leading to the formation of zigzag chains propagating along [010]. These chains are linked via C-H···N hydrogen bonds forming undulating two-dimensional networks extending in the a and b directions (Fig. 2 and Table 1).

### Experimental

A mixture of 4-formyl triphenylamine (0.19 g, 0.7 mmol), 1,10-phenanthroline-5,6-dione (0.15 g, 0.7 mmol), ammonium acetate (1.15 g, 15 mmol) and 15 mL acetic acid were heated and refluxed for 4 h, then cooled to room temperature. 30 mL water was added and a saturated K_2_CO_3_ solution was added slowly to adjust the pH to 7–8. A yellow precipitate was generated gradually, suction filtered and washed with water three times, and recrystallized from ethanol to give a pale-yellow bock-like crystals (0.28 g; Yield 85%) Spectroscopic details for the title compound are available in the archived CIF.

### Refinement

The amine H atoms were located in a difference Fourier map and freely refined. The OH and C-bound H atoms were placed in geometrically idealized positions and constrained to ride on their parent atoms: O—H = 0.82 Å, C—H = 0.93–0.97 Å, with *U*_iso_(H) = 1.5*U*_eq_(atoms C33 and O1), and = 1.2*U*_eq_(C) for other H atoms.

### Crystal data

**Table:**

C_32_H_21_N_4_·C_2_H_6_O	*F*(000) = 1068
*M**_r_* = 507.60	*D*_x_ = 1.202 Mg m^−^^3^
Monoclinic, *P*2_1_/*c*	Mo *K*α radiation, λ = 0.71073 Å
Hall symbol: -P 2ybc	Cell parameters from 6506 reflections
*a* = 9.716 (4) Å	θ = 2.4–25.0°
*b* = 10.690 (4) Å	µ = 0.07 mm^−^^1^
*c* = 27.017 (10) Å	*T* = 296 K
β = 92.317 (4)°	Block, yellow
*V* = 2803.8 (18) Å^3^	0.20 × 0.20 × 0.10 mm
*Z* = 4	

### Data collection

**Table:**

Bruker APEXII CCD diffractometer	5216 independent reflections
Radiation source: fine-focus sealed tube	3870 reflections with *I* > 2σ(*I*)
Graphite monochromator	*R*_int_ = 0.024
φ and ω scans	θ_max_ = 25.5°, θ_min_ = 1.5°
Absorption correction: multi-scan (*SADABS*; Bruker, 2007)	*h* = −11→11
*T*_min_ = 0.985, *T*_max_ = 0.993	*k* = −12→12
20210 measured reflections	*l* = −32→32

### Refinement

**Table:**

Refinement on *F*^2^	Primary atom site location: structure-invariant direct methods
Least-squares matrix: full	Secondary atom site location: difference Fourier map
*R*[*F*^2^ > 2σ(*F*^2^)] = 0.065	Hydrogen site location: inferred from neighbouring sites
*wR*(*F*^2^) = 0.210	H atoms treated by a mixture of independent and constrained refinement
*S* = 1.02	*w* = 1/[σ^2^(*F*_o_^2^) + (0.1148*P*)^2^ + 1.3126*P*] where *P* = (*F*_o_^2^ + 2*F*_c_^2^)/3
5216 reflections	(Δ/σ)_max_ = 0.020
340 parameters	Δρ_max_ = 0.51 e Å^−^^3^
6 restraints	Δρ_min_ = −0.50 e Å^−^^3^

### Special details

**Table:**

Experimental. Spectroscopic details for the title compound: ^1^H NMR (400 MHz, DMSO-d6) δ (p.p.m.): 7.12–7.15 (m, 8H), 7.38 (t, J=8.0 Hz, 4H), 7.82 (q, J=4.2 Hz, 2H), 8.17 (d, J=4.0 Hz, 2H), 8.89–8.92 (m, 2H), 9.01–9.02 (m, 2H). ^13^C NMR (150 MHz, DMSO-d6) δ (p.p.m.) 151.0, 148.2, 147.4, 146.7, 143.4, 129.7, 129.5, 127.5, 124.6, 123.9, 123.7, 123.1, 122.05. IR (KBr, cm^-1^): 3422(m), 3030(s), 1607(versus), 1485(versus), 1331(s), 1286(s), 1137(w), 739(s), 700(s). MS: m/z (%)= 464.12(M+, 100%).
Geometry. Bond distances, angles etc. have been calculated using the rounded fractional coordinates. All su's are estimated from the variances of the (full) variance-covariance matrix. The cell esds are taken into account in the estimation of distances, angles and torsion angles
Refinement. Refinement of *F*^2^ against ALL reflections. The weighted *R*-factor *wR* and goodness of fit *S* are based on *F*^2^, conventional *R*-factors *R* are based on *F*, with *F* set to zero for negative *F*^2^. The threshold expression of *F*^2^ > σ(*F*^2^) is used only for calculating *R*-factors(gt) *etc*. and is not relevant to the choice of reflections for refinement. *R*-factors based on *F*^2^ are statistically about twice as large as those based on *F*, and *R*- factors based on ALL data will be even larger.

### Fractional atomic coordinates and isotropic or equivalent isotropic displacement parameters (Å^2^)

**Table:**

	*x*	*y*	*z*	*U*_iso_*/*U*_eq_	
N1	0.4844 (2)	−0.1863 (2)	0.29749 (8)	0.0636 (8)	
N2	0.2262 (2)	−0.28229 (19)	0.29875 (7)	0.0536 (7)	
N3	0.3274 (2)	0.04565 (18)	0.14710 (7)	0.0474 (6)	
N4	0.0133 (2)	0.3171 (2)	−0.04486 (8)	0.0693 (8)	
C1	0.6091 (3)	−0.1386 (3)	0.29806 (11)	0.0795 (11)	
C2	0.6544 (3)	−0.0539 (3)	0.26334 (12)	0.0786 (11)	
C3	0.5652 (3)	−0.0170 (3)	0.22597 (9)	0.0607 (8)	
C4	0.4309 (2)	−0.0651 (2)	0.22369 (8)	0.0473 (7)	
C5	0.3260 (2)	−0.0335 (2)	0.18724 (8)	0.0442 (7)	
C6	0.1942 (2)	−0.0808 (2)	0.18772 (8)	0.0447 (7)	
C7	0.1549 (2)	−0.1673 (2)	0.22489 (8)	0.0454 (7)	
C8	0.2546 (2)	−0.2019 (2)	0.26137 (8)	0.0450 (7)	
C9	0.3942 (2)	−0.1511 (2)	0.26082 (8)	0.0471 (7)	
C10	0.0223 (3)	−0.2189 (3)	0.22706 (10)	0.0622 (9)	
C11	−0.0038 (3)	−0.3001 (3)	0.26445 (11)	0.0718 (10)	
C12	0.1002 (3)	−0.3279 (3)	0.29922 (10)	0.0629 (9)	
C13	0.11393 (19)	−0.03287 (18)	0.14906 (7)	0.0347 (6)	
C14	0.1974 (2)	0.0425 (2)	0.12546 (8)	0.0469 (7)	
C15	0.1551 (2)	0.1150 (2)	0.08140 (8)	0.0466 (7)	
C16	0.2373 (3)	0.2055 (3)	0.06061 (10)	0.0627 (9)	
C17	0.1902 (3)	0.2714 (3)	0.01937 (10)	0.0687 (10)	
C18	0.0619 (3)	0.2493 (2)	−0.00245 (9)	0.0568 (8)	
C19	−0.0198 (3)	0.1581 (2)	0.01814 (9)	0.0568 (8)	
C20	0.0266 (2)	0.0926 (2)	0.05941 (9)	0.0554 (8)	
C21	0.10408 (15)	0.33915 (15)	−0.08378 (4)	0.0739 (11)	
C22	0.19694 (16)	0.24825 (19)	−0.09791 (7)	0.0840 (13)	
C23	0.28428 (16)	0.2720 (3)	−0.13631 (8)	0.1121 (16)	
C24	0.2788 (2)	0.3867 (3)	−0.16057 (7)	0.1252 (12)	
C25	0.1859 (2)	0.4776 (2)	−0.14644 (8)	0.152 (3)	
C26	0.0986 (2)	0.45382 (15)	−0.10804 (7)	0.1127 (18)	
C27	−0.12987 (17)	0.34159 (19)	−0.05179 (6)	0.0672 (10)	
C28	−0.19577 (14)	0.3242 (2)	−0.09755 (8)	0.0911 (14)	
C29	−0.3348 (5)	0.3488 (5)	−0.10367 (17)	0.1232 (19)	
C30	−0.4097 (5)	0.3866 (5)	−0.0651 (2)	0.132 (2)	
C31	−0.3455 (4)	0.4019 (4)	−0.01915 (16)	0.1133 (17)	
C32	−0.2059 (4)	0.3818 (3)	−0.01277 (12)	0.0864 (12)	
O1	0.55771 (19)	0.1730 (2)	0.11618 (7)	0.0698 (7)	
C33	0.6124 (4)	0.0588 (5)	0.04600 (15)	0.1228 (19)	
C34	0.6648 (4)	0.1264 (5)	0.08975 (13)	0.1040 (16)	
H1	0.67110	−0.16310	0.32340	0.0950*	
H2	0.74390	−0.02300	0.26550	0.0940*	
H3	0.59310	0.03960	0.20220	0.0730*	
H3N	0.404 (3)	0.087 (3)	0.1347 (10)	0.069 (8)*	
H10	−0.04630	−0.19810	0.20350	0.0750*	
H11	−0.09040	−0.33630	0.26660	0.0860*	
H12	0.07980	−0.38260	0.32470	0.0760*	
H16	0.32460	0.22180	0.07450	0.0750*	
H17	0.24640	0.33210	0.00600	0.0820*	
H19	−0.10660	0.14110	0.00400	0.0680*	
H20	−0.02980	0.03200	0.07280	0.0660*	
H22	0.20060	0.17150	−0.08170	0.1010*	
H23	0.34640	0.21120	−0.14580	0.1340*	
H24	0.33720	0.40260	−0.18630	0.1500*	
H25	0.18220	0.55430	−0.16270	0.1830*	
H26	0.03640	0.51460	−0.09860	0.1350*	
H28	−0.14650	0.29610	−0.12420	0.1090*	
H29	−0.37800	0.33920	−0.13480	0.1480*	
H30	−0.50350	0.40210	−0.06960	0.1580*	
H31	−0.39650	0.42580	0.00770	0.1360*	
H32	−0.16250	0.39550	0.01810	0.1040*	
H1O1	0.58880	0.21010	0.14080	0.1050*	
H33A	0.54660	−0.00260	0.05560	0.1850*	
H33B	0.68740	0.01800	0.03050	0.1850*	
H33C	0.56890	0.11660	0.02310	0.1850*	
H34A	0.72020	0.07050	0.11070	0.1250*	
H34B	0.72300	0.19470	0.07970	0.1250*	

### Atomic displacement parameters (Å^2^)

**Table:**

	*U*^11^	*U*^22^	*U*^33^	*U*^12^	*U*^13^	*U*^23^
N1	0.0568 (13)	0.0751 (15)	0.0579 (12)	−0.0092 (11)	−0.0105 (10)	0.0180 (11)
N2	0.0557 (12)	0.0555 (12)	0.0498 (11)	0.0006 (9)	0.0064 (9)	0.0116 (9)
N3	0.0465 (11)	0.0519 (11)	0.0440 (10)	−0.0024 (9)	0.0030 (8)	0.0078 (8)
N4	0.0756 (16)	0.0745 (15)	0.0568 (13)	0.0038 (12)	−0.0105 (11)	0.0201 (11)
C1	0.0643 (18)	0.100 (2)	0.0722 (18)	−0.0165 (16)	−0.0235 (14)	0.0295 (17)
C2	0.0559 (16)	0.097 (2)	0.0814 (19)	−0.0211 (15)	−0.0158 (14)	0.0271 (17)
C3	0.0530 (14)	0.0703 (16)	0.0584 (14)	−0.0112 (12)	−0.0029 (11)	0.0142 (12)
C4	0.0472 (12)	0.0501 (13)	0.0446 (11)	0.0007 (10)	0.0030 (9)	0.0015 (10)
C5	0.0472 (12)	0.0455 (12)	0.0402 (11)	0.0017 (9)	0.0044 (9)	0.0018 (9)
C6	0.0459 (12)	0.0442 (12)	0.0443 (11)	0.0012 (9)	0.0043 (9)	0.0014 (9)
C7	0.0453 (12)	0.0473 (12)	0.0437 (11)	0.0015 (9)	0.0038 (9)	0.0019 (9)
C8	0.0486 (12)	0.0433 (12)	0.0433 (11)	0.0016 (9)	0.0057 (9)	0.0017 (9)
C9	0.0497 (13)	0.0487 (13)	0.0426 (11)	0.0006 (10)	−0.0006 (9)	0.0038 (9)
C10	0.0509 (14)	0.0713 (17)	0.0640 (15)	−0.0073 (12)	−0.0020 (11)	0.0173 (13)
C11	0.0546 (15)	0.083 (2)	0.0779 (19)	−0.0134 (14)	0.0052 (13)	0.0241 (15)
C12	0.0572 (15)	0.0674 (17)	0.0646 (15)	−0.0058 (12)	0.0074 (12)	0.0211 (13)
C13	0.0322 (10)	0.0375 (10)	0.0342 (9)	0.0020 (8)	0.0003 (7)	0.0060 (8)
C14	0.0487 (13)	0.0488 (13)	0.0432 (11)	0.0054 (10)	0.0011 (9)	−0.0005 (9)
C15	0.0484 (12)	0.0484 (13)	0.0430 (11)	0.0036 (10)	0.0007 (9)	0.0029 (9)
C16	0.0581 (15)	0.0708 (17)	0.0584 (15)	−0.0095 (13)	−0.0087 (12)	0.0167 (12)
C17	0.0700 (17)	0.0709 (18)	0.0641 (16)	−0.0164 (14)	−0.0090 (13)	0.0245 (14)
C18	0.0645 (15)	0.0572 (14)	0.0482 (12)	0.0040 (12)	−0.0043 (11)	0.0084 (11)
C19	0.0510 (14)	0.0660 (16)	0.0529 (13)	0.0001 (12)	−0.0055 (11)	0.0073 (12)
C20	0.0530 (14)	0.0604 (15)	0.0527 (13)	−0.0027 (11)	0.0019 (11)	0.0089 (11)
C21	0.090 (2)	0.081 (2)	0.0492 (14)	−0.0295 (17)	−0.0172 (14)	0.0199 (14)
C22	0.076 (2)	0.107 (3)	0.0690 (18)	−0.0173 (19)	0.0048 (15)	0.0109 (18)
C23	0.089 (2)	0.168 (4)	0.079 (2)	−0.035 (3)	0.0010 (19)	0.008 (2)
C24	0.127 (2)	0.136 (2)	0.113 (2)	−0.0215 (18)	0.0084 (17)	0.0141 (18)
C25	0.199 (5)	0.160 (5)	0.096 (3)	−0.080 (4)	−0.023 (3)	0.079 (3)
C26	0.165 (4)	0.094 (3)	0.077 (2)	−0.031 (3)	−0.020 (2)	0.036 (2)
C27	0.0789 (19)	0.0555 (15)	0.0655 (16)	0.0121 (13)	−0.0188 (14)	0.0001 (13)
C28	0.095 (2)	0.101 (3)	0.075 (2)	0.013 (2)	−0.0249 (18)	−0.0162 (18)
C29	0.105 (3)	0.154 (4)	0.106 (3)	0.024 (3)	−0.051 (3)	−0.034 (3)
C30	0.098 (3)	0.148 (4)	0.145 (4)	0.048 (3)	−0.041 (3)	−0.044 (3)
C31	0.104 (3)	0.119 (3)	0.115 (3)	0.054 (3)	−0.019 (2)	−0.031 (3)
C32	0.101 (2)	0.078 (2)	0.078 (2)	0.0311 (19)	−0.0226 (18)	−0.0157 (16)
O1	0.0663 (12)	0.0912 (14)	0.0521 (10)	−0.0245 (10)	0.0053 (8)	−0.0148 (9)
C33	0.098 (3)	0.174 (4)	0.097 (3)	−0.016 (3)	0.011 (2)	−0.060 (3)
C34	0.074 (2)	0.162 (4)	0.077 (2)	−0.028 (2)	0.0170 (17)	−0.036 (2)

### Geometric parameters (Å, º)

**Table:**

O1—C34	1.379 (4)	C23—C24	1.390 (4)
O1—H1O1	0.8200	C24—C25	1.390 (3)
N1—C9	1.349 (3)	C25—C26	1.390 (3)
N1—C1	1.314 (4)	C27—C32	1.380 (4)
N2—C8	1.363 (3)	C27—C28	1.382 (3)
N2—C12	1.318 (4)	C28—C29	1.380 (5)
N3—C14	1.371 (3)	C29—C30	1.357 (7)
N3—C5	1.376 (3)	C30—C31	1.376 (7)
N4—C27	1.420 (3)	C31—C32	1.377 (6)
N4—C18	1.420 (3)	C1—H1	0.9300
N4—C21	1.419 (2)	C2—H2	0.9300
N3—H3N	0.94 (3)	C3—H3	0.9300
C1—C2	1.388 (4)	C10—H10	0.9300
C2—C3	1.362 (4)	C11—H11	0.9300
C3—C4	1.402 (4)	C12—H12	0.9300
C4—C9	1.417 (3)	C16—H16	0.9300
C4—C5	1.429 (3)	C17—H17	0.9300
C5—C6	1.377 (3)	C19—H19	0.9300
C6—C7	1.428 (3)	C20—H20	0.9300
C6—C13	1.377 (3)	C22—H22	0.9300
C7—C10	1.405 (4)	C23—H23	0.9300
C7—C8	1.403 (3)	C24—H24	0.9300
C8—C9	1.462 (3)	C25—H25	0.9300
C10—C11	1.363 (4)	C26—H26	0.9300
C11—C12	1.384 (4)	C28—H28	0.9300
C13—C14	1.325 (3)	C29—H29	0.9300
C14—C15	1.465 (3)	C30—H30	0.9300
C15—C20	1.382 (3)	C31—H31	0.9300
C15—C16	1.388 (4)	C32—H32	0.9300
C16—C17	1.381 (4)	C33—C34	1.459 (6)
C17—C18	1.378 (4)	C33—H33A	0.9600
C18—C19	1.388 (4)	C33—H33B	0.9600
C19—C20	1.377 (3)	C33—H33C	0.9600
C21—C22	1.390 (2)	C34—H34A	0.9700
C21—C26	1.390 (2)	C34—H34B	0.9700
C22—C23	1.390 (3)		
			
C34—O1—H1O1	109.00	C27—C28—C29	119.9 (2)
C1—N1—C9	118.1 (2)	C28—C29—C30	121.2 (4)
C8—N2—C12	117.2 (2)	C29—C30—C31	119.4 (4)
C5—N3—C14	106.43 (18)	C30—C31—C32	120.2 (4)
C18—N4—C27	119.5 (2)	C27—C32—C31	120.5 (3)
C21—N4—C27	120.42 (17)	C2—C1—H1	118.00
C18—N4—C21	119.09 (19)	N1—C1—H1	118.00
C5—N3—H3N	127.2 (17)	C1—C2—H2	121.00
C14—N3—H3N	126.1 (17)	C3—C2—H2	121.00
N1—C1—C2	124.2 (3)	C4—C3—H3	120.00
C1—C2—C3	118.7 (3)	C2—C3—H3	120.00
C2—C3—C4	119.3 (3)	C7—C10—H10	121.00
C3—C4—C9	117.9 (2)	C11—C10—H10	121.00
C5—C4—C9	116.64 (18)	C10—C11—H11	120.00
C3—C4—C5	125.5 (2)	C12—C11—H11	120.00
N3—C5—C6	105.78 (18)	C11—C12—H12	118.00
C4—C5—C6	123.0 (2)	N2—C12—H12	118.00
N3—C5—C4	131.23 (19)	C17—C16—H16	120.00
C7—C6—C13	127.94 (18)	C15—C16—H16	120.00
C5—C6—C13	110.77 (19)	C16—C17—H17	119.00
C5—C6—C7	121.29 (19)	C18—C17—H17	119.00
C6—C7—C10	123.7 (2)	C20—C19—H19	120.00
C8—C7—C10	118.4 (2)	C18—C19—H19	120.00
C6—C7—C8	117.89 (18)	C15—C20—H20	119.00
N2—C8—C7	122.14 (18)	C19—C20—H20	119.00
C7—C8—C9	120.63 (19)	C23—C22—H22	120.00
N2—C8—C9	117.23 (18)	C21—C22—H22	120.00
N1—C9—C4	121.75 (19)	C22—C23—H23	120.00
C4—C9—C8	120.56 (18)	C24—C23—H23	120.00
N1—C9—C8	117.67 (19)	C23—C24—H24	120.00
C7—C10—C11	118.6 (3)	C25—C24—H24	120.00
C10—C11—C12	119.2 (3)	C26—C25—H25	120.00
N2—C12—C11	124.5 (3)	C24—C25—H25	120.00
C6—C13—C14	104.62 (17)	C25—C26—H26	120.00
C13—C14—C15	123.75 (18)	C21—C26—H26	120.00
N3—C14—C15	123.84 (18)	C27—C28—H28	120.00
N3—C14—C13	112.40 (19)	C29—C28—H28	120.00
C14—C15—C16	123.4 (2)	C28—C29—H29	119.00
C14—C15—C20	118.46 (19)	C30—C29—H29	119.00
C16—C15—C20	118.1 (2)	C31—C30—H30	120.00
C15—C16—C17	120.3 (3)	C29—C30—H30	120.00
C16—C17—C18	121.5 (3)	C30—C31—H31	120.00
C17—C18—C19	118.1 (2)	C32—C31—H31	120.00
N4—C18—C17	121.6 (2)	C31—C32—H32	120.00
N4—C18—C19	120.3 (2)	C27—C32—H32	120.00
C18—C19—C20	120.5 (2)	O1—C34—C33	110.6 (3)
C15—C20—C19	121.4 (2)	C34—C33—H33A	110.00
N4—C21—C22	121.14 (16)	C34—C33—H33B	109.00
C22—C21—C26	120.00 (14)	C34—C33—H33C	109.00
N4—C21—C26	118.86 (16)	H33A—C33—H33B	109.00
C21—C22—C23	120.0 (2)	H33A—C33—H33C	109.00
C22—C23—C24	120.0 (2)	H33B—C33—H33C	109.00
C23—C24—C25	119.99 (18)	O1—C34—H34A	109.00
C24—C25—C26	120.0 (2)	O1—C34—H34B	110.00
C21—C26—C25	120.03 (16)	C33—C34—H34A	110.00
N4—C27—C28	120.60 (16)	C33—C34—H34B	110.00
C28—C27—C32	118.8 (2)	H34A—C34—H34B	108.00
N4—C27—C32	120.6 (2)		
			
C9—N1—C1—C2	0.1 (4)	C6—C7—C8—N2	179.0 (2)
C1—N1—C9—C8	−178.3 (2)	C10—C7—C8—N2	−0.7 (3)
C1—N1—C9—C4	−0.1 (3)	C6—C7—C8—C9	−0.4 (3)
C12—N2—C8—C7	0.6 (3)	C6—C7—C10—C11	−179.6 (2)
C12—N2—C8—C9	179.9 (2)	C8—C7—C10—C11	0.1 (4)
C8—N2—C12—C11	0.3 (4)	C10—C7—C8—C9	179.9 (2)
C5—N3—C14—C15	179.6 (2)	N2—C8—C9—C4	−178.8 (2)
C14—N3—C5—C4	−178.7 (2)	C7—C8—C9—N1	178.8 (2)
C14—N3—C5—C6	0.1 (2)	N2—C8—C9—N1	−0.6 (3)
C5—N3—C14—C13	0.0 (2)	C7—C8—C9—C4	0.6 (3)
C27—N4—C21—C26	49.8 (3)	C7—C10—C11—C12	0.7 (4)
C27—N4—C18—C17	−148.4 (2)	C10—C11—C12—N2	−0.9 (5)
C27—N4—C21—C22	−129.95 (19)	C6—C13—C14—C15	−179.7 (2)
C18—N4—C21—C26	−141.73 (19)	C6—C13—C14—N3	−0.1 (2)
C21—N4—C18—C17	43.0 (3)	N3—C14—C15—C16	−8.0 (4)
C18—N4—C27—C28	−135.0 (2)	N3—C14—C15—C20	172.2 (2)
C18—N4—C27—C32	44.3 (3)	C13—C14—C15—C20	−8.3 (3)
C18—N4—C21—C22	38.6 (3)	C13—C14—C15—C16	171.5 (2)
C21—N4—C27—C28	33.5 (3)	C16—C15—C20—C19	−0.3 (3)
C27—N4—C18—C19	31.7 (3)	C20—C15—C16—C17	0.6 (4)
C21—N4—C18—C19	−136.9 (2)	C14—C15—C20—C19	179.5 (2)
C21—N4—C27—C32	−147.3 (2)	C14—C15—C16—C17	−179.2 (2)
N1—C1—C2—C3	−0.1 (5)	C15—C16—C17—C18	−0.4 (4)
C1—C2—C3—C4	0.1 (4)	C16—C17—C18—N4	180.0 (3)
C2—C3—C4—C5	179.0 (3)	C16—C17—C18—C19	−0.1 (4)
C2—C3—C4—C9	−0.1 (4)	C17—C18—C19—C20	0.4 (4)
C9—C4—C5—C6	1.2 (3)	N4—C18—C19—C20	−179.7 (2)
C5—C4—C9—C8	−1.0 (3)	C18—C19—C20—C15	−0.2 (4)
C3—C4—C5—N3	0.7 (4)	N4—C21—C22—C23	179.69 (17)
C3—C4—C5—C6	−177.9 (2)	C26—C21—C22—C23	0.0 (3)
C3—C4—C9—N1	0.1 (3)	N4—C21—C26—C25	−179.69 (18)
C3—C4—C9—C8	178.2 (2)	C22—C21—C26—C25	0.0 (3)
C5—C4—C9—N1	−179.1 (2)	C21—C22—C23—C24	0.0 (3)
C9—C4—C5—N3	179.8 (2)	C22—C23—C24—C25	0.0 (3)
C4—C5—C6—C7	−1.1 (3)	C23—C24—C25—C26	0.1 (3)
N3—C5—C6—C13	−0.2 (2)	C24—C25—C26—C21	0.0 (3)
C4—C5—C6—C13	178.7 (2)	N4—C27—C28—C29	−179.8 (3)
N3—C5—C6—C7	−179.94 (19)	C32—C27—C28—C29	0.9 (4)
C13—C6—C7—C8	−179.1 (2)	N4—C27—C32—C31	−178.0 (3)
C5—C6—C7—C8	0.6 (3)	C28—C27—C32—C31	1.2 (4)
C7—C6—C13—C14	179.9 (2)	C27—C28—C29—C30	−1.9 (6)
C5—C6—C7—C10	−179.7 (2)	C28—C29—C30—C31	0.7 (8)
C5—C6—C13—C14	0.2 (2)	C29—C30—C31—C32	1.5 (7)
C13—C6—C7—C10	0.5 (4)	C30—C31—C32—C27	−2.5 (6)

### Hydrogen-bond geometry (Å, º)

**Table:**

*D*—H···*A*	*D*—H	H···*A*	*D*···*A*	*D*—H···*A*
N3—H3*N*···O1	0.94 (3)	1.84 (3)	2.777 (3)	175 (2)
O1—H1*O*1···N1^i^	0.82	2.15	2.819 (3)	139
O1—H1*O*1···N2^i^	0.82	2.38	3.087 (3)	145
C16—H16···O1	0.93	2.54	3.419 (4)	157
C3—H3···N2^i^	0.93	2.59	3.310 (4)	135

Symmetry code: (i) −*x*+1, *y*+1/2, −*z*+1/2.
